# Mitochondria-targeted curcumin loaded CTPP–PEG–PCL self-assembled micelles for improving liver fibrosis therapy†

**DOI:** 10.1039/d0ra09589c

**Published:** 2021-01-28

**Authors:** Liqiao Zhang, Xiuhua Pan, Lixing Xu, Linlin Zhang, Haiqin Huang

**Affiliations:** Department of Pharmacy, Chengdu Women's and Children's Central Hospital, School of Medicine, University of Electronic Science and Technology of China Chengdu 611731 PR China; College of Pharmacy, Nantong University Nantong 226001 PR China huanghaiqincpu@163.com; Nanjing Chia Tai Tianqing Pharmaceutical CO., Ltd Nanjing 210000 PR China zll15366099319@163.com; Key Laboratory of Modern Chinese Medicines, China Pharmaceutical University Nanjing 210009 China

## Abstract

Liver fibrosis, originating from activated hepatic stellate cells (HSCs), is receiving much attention in the treatment of clinical liver disease. In this study, mitochondria-targeted curcumin (Cur) loaded 3-carboxypropyl-triphenylphosphonium bromide–poly(ethylene glycol)–poly(ε-caprolactone) (CTPP–PEG–PCL) micelles were constructed to prolong the systemic circulation of Cur, improve the bioavailability of Cur and play a precise role in anti-fibrosis. The prepared Cur–CTPP–PEG–PCL micelles with a spherical shape had satisfactory dispersion, low critical micelle concentration (CMC) and high encapsulation efficiency (92.88%). The CTPP modification endowed good endosomal escape ability to the CTPP–PEG–PCL micelles, and micelles could be selectively accumulated in mitochondria, thereby inducing the enhanced cell proliferation inhibition of HSC-T6 cells. Mitochondrial Membrane Potential (MMP) was greatly reduced due to the mitochondrial-targeting of Cur. Moreover, the system circulation of Cur was extended and bioavailability was significantly enhanced *in vivo*. As expected, Cur loaded CTPP–PEG–PCL micelles were more effective in improving liver fibrosis compared with Cur and Cur–mPEG–PCL micelles. In conclusion, the Cur–CTPP–PEG–PCL based micelles can be a potential candidate for liver fibrosis treatment in future clinical applications.

## Abstract 2

A mitochondria-targeting micelle system based on CTPP–PEG–PCL polymer was designed to deliver curcumin to active HSC-T6 cells and prolong the systemic circulation and bioavailability of curcumin *in vivo* for effective treatment of liver fibrosis.

## Graphical abstract

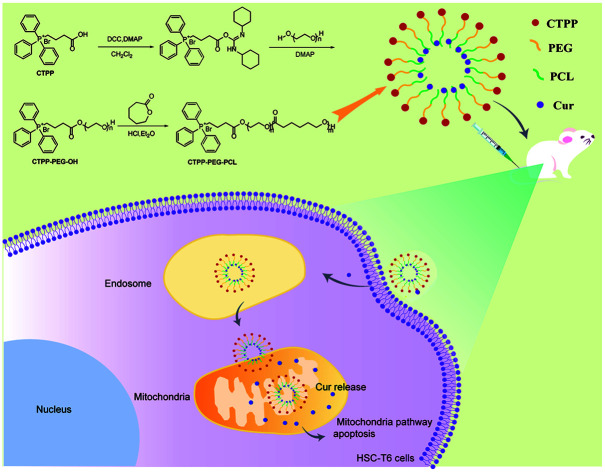

## 1. Introduction

Liver fibrosis is a compensatory response of tissue self-repair after persistent liver damage caused by various pathogenic factors, and it is the most common histopathological change in the development of various chronic liver diseases.^1^ Liver fibrosis is a reversible process and will be reversed if effective treatment is given.^2^ The main manifestations of liver fibrosis are the unbalanced generation and degradation of the extracellular matrix (ECM) based on type I and IV collagen, and abnormal deposition of fibrous connective tissue, which will damage the normal structure and function of the liver.^3,4^ Experimental and clinical data have demonstrated that the massive activation and proliferation of hepatic stellate cells (HSC) are important factors affecting the development of liver fibrosis.^5,6^ Conventional HSC is usually in a static state, and the cytoplasm is rich in vitamin A lipids droplets. Generally, HSC in the resting state (under normal conditions) does not synthesize or only synthesizes a small amount of ECM. However, when pathogenic factors continue to damage the liver, the resting HSC will activate proliferation and transform into myofibroblast-like cells (MFB).^7^ Research has shown that HSC activation is mainly manifested by increased collagen expression, large loss of intracellular lipid droplets and significantly enhanced cell shrinkage and migration capabilities, which will lead to excessive secretion of ECM and deposition in the liver disease space and hepatocyte necrosis area, which will lead to the occurrence and development of liver fibrosis.^8,9^ Activated HSC is the main source of ECM components in liver fibrosis and plays an important effect on regulating the degradation of ECM.^2^ Thence, the key point to preventing liver fibrosis is to inhibit the activation of HSCs.^10–12^ Clinical researchers are hunt for effective means for the treatment of liver fibrosis by reducing the number of activated HSCs in the liver to reduce the generation of ECM. Research on delivery systems for HSCs has important clinical significance.

Curcumin is a naturally-occurring polyphenol phytochemical, which possess various pharmacological activities such as antioxidant, anti-inflammatory, and protecting liver function.^13–15^ Researches have shown that curcumin can significantly improve liver fibrosis, including inhibiting HSC activation,^16,17^ inhibiting the conversion of activated HSC into myofibroblasts,^18^ restoring the lipid droplet content in HSC,^19^ and promoting HSC cell apoptosis.^20^ Curcumin exerting satisfactory effects on liver injury in animal models had proven by Park *et al.*^21^ Bruck *et al.*^22^ demonstrated that curcumin inhibited the liver fibrosis by reducing oxidative stress and inhibiting the activation of HSC. However, pharmacokinetic studies *in vivo* have proven that curcumin has poor bioavailability, which limit the translation to clinically treatment.^23^ The main challenge of curcumin is to solve its shortcomings such as poor solubility in aqueous solutions and poor bioavailability, which limit its clinical application.^24^ In order to overcome the limitation of curcumin, extensive attempts have been made such as polymer micelles, nanoparticles, liposomes, and hydrogels.^25–28^ However, the application of these delivery system has been further restricted due to the lack of selectivity and high toxicity to normal cells. Therefore, rummaging a suitable delivery vehicle is a necessary prerequisite for curcumin to be applied in clinical.

Mitochondria, which produce adenosine triphosphate (ATP), is involved in the cell signal transduction process from cell cycle, cell differentiation to apoptosis signal.^29^ Mitochondria play an important role in inducing cell apoptosis by activating the pro-apoptotic protein Bcl-2. This may lead to a decrease in the mitochondrial membrane potential and the release of cytochrome c from the mitochondria into the cytoplasm, leading to initial cell apoptosis.^30^ Therefore, mitochondrial targeted therapy is considered as a promising strategy for the treatment of diseases.^31^ The mitochondrial-mediated apoptosis pathway in hepatic fibroblasts is inhibited due to the overexpression of anti-apoptotic proteins (such as Bcl-2) and the blocking of mitochondrial membrane permeability.^32^ Studies demonstrated that curcumin could activate the mitochondrial apoptosis pathway by regulating the level of Bcl-2 and promote the cell apoptosis.^33,34^ Therefore, constructing an effective delivery system to precise deliver curcumin to the mitochondria may greatly improve the clinical efficacy.

To date, various mitochondrial drug delivery systems have been developed for mitochondria-targeted drug delivery.^35^ Cationic lipophilic material triphenylphosphonium (TPP), possess the ability of penetrate the mitochondrial membrane, is often used to deliver drugs and polymer materials into mitochondrial by overcoming the barrier hinders of cell membranes and mitochondrial membranes.^36,37^ Poly(ethylene glycol)–poly(ε-caprolactone) (PEG–PCL) is an amphiphilic polymer that can self-assemble to form nano-micelles after being dissolved in water.^38^ At present, many researches have confirmed that PEG–PCL had a well enhanced permeability and retention (EPR) effect in tumor tissues and could be accumulated in tumor cells by passive targeting.^39^

Based on the above situation, we intended to modify mPEG–PCL polymer with lipophilic cationic 3-carboxypropyl-triphenylphosphonium bromide (CTPP), encapsulating Cur into self-assembled micelles effectively, thus might deliver Cur to mitochondria accurately and promote the apoptosis of HSC-T6 cell. The constructed delivery system was expected to improve the bioavailability of Cur *in vivo* and effectively treat liver fibrosis, thereby providing an alternative candidate for liver fibrosis in clinical application.

## 2. Materials and methods

### 2.1 Materials

3-Carboxypropyl-triphenylphosphonium bromide (CTPP) was obtained from Adamas Reagent Co., Ltd (Shanghai, China). Polyethylene glycol (PEG), ε-caprolactone was obtained from Daigang Biomaterial (Shandong, China). *N*,*N*′-Dicyclohexylcarbodimide (DCC) and dimethylaminopyridine (DMAP) were obtained from Sinopharm Chemical Reagent Co., Ltd (Shanghai, China). Curcumin was purchased from Adamas Reagent Co., Ltd (Shanghai, China). LysoTracker Red, 4% paraformaldehyde and Hoechst 33258 were obtained from Beyotime Biotechnology (Shanghai, China). RPMI-1640 medium and 3-(4,5-dimethylthiazol-2-yl)-2,5-diphenyltetrazolium bromide (MTT) were obtained from KeyGen Biotech (Nanjing, China). Fetal bovine serum (FBS) was purchased from Gibco (Hangzhou, China). All the other chemicals and reagents were analytic grade.

### 2.2 Cell culture

Hepatic Stellate Cells (HSC-T6) and Alpha Mouse Liver 12 (AML- 12) used in the study were purchased from the American Type Culture Collection (ATCC) and cultured in RPMI 1640 medium (pH = 7.4 ± 0.2) supplemented with 10% FBS and penicillin-streptomycin (100 IU mL^−1^ to 100 μg mL^−1^) under 5% CO_2_ atmosphere at 37 °C.

### 2.3 Animals

SD rat (200 ± 20 g) and Balb/c mice were obtained from Qinglong Mountain Animal Farm (Nanjing, China). Balb/c mice (18 ± 2 g) were also purchased from Qinglong Mountain Animal Breeding Farm (Nanjing, China). All animal procedures were performed in accordance with the Guidelines for Care and Use of Laboratory Animals of Nantong University and experiments were approved by the Animal Ethics Committee of Nantong University (Approval number: NTU201811).

### 2.4 Synthesis and characterization of CTPP–PEG–PCL copolymer

**Scheme 1:**
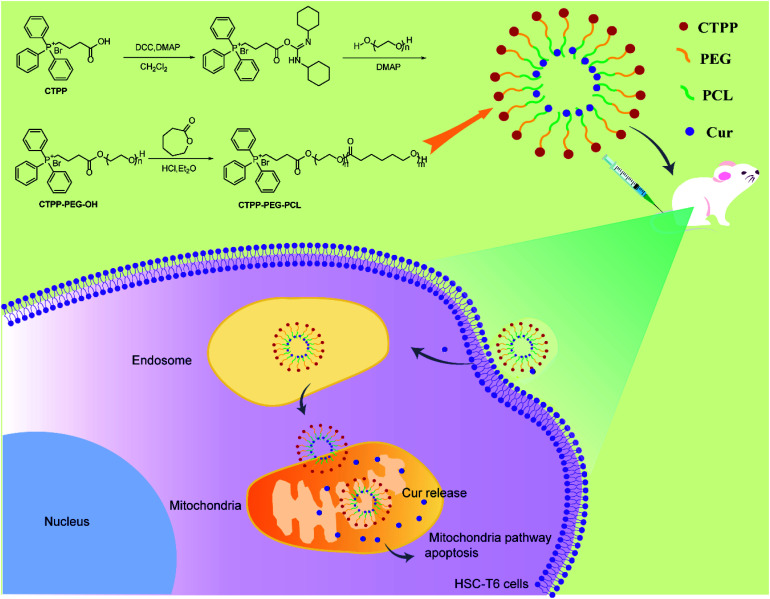
Illustration of Cur-load CTPP–PEG–PCL micelles for anti-fibrosis treatment. CTPP–PEG–PCL was synthesized according to the route and encapsulated Cur by self-assemble of micelles. Cur–CTPP–PEG–PCL micelles were passive uptake by liver fibrosis cells after intravenous injection to liver-fibrosis mouse. Cur–CTPP–PEG–PCL micelles could escape from endosome and enter in mitochondria with the assistance with CTPP, thus inducing apoptosis with the Cur release.

CTPP–PEG–PCL copolymer was synthesized according to the following two steps (Scheme 1). First, CTPP–PEG–OH was synthesized by using DCC as the condensing agent and DMAP as the catalyst. Firstly, 0.2 mg CTPP, 0.2 mg DCC and 0.02 mg DMAP were dissolved in chloroform at a molar ratio of 2.5 : 5.5 : 1 and PEG was added to the mixture in batches, and finally stirred for another 96 h under an N_2_ atmosphere. Subsequently, the insoluble dicyclohexylurea was removed by filtration, and the chloroform was removed by rotary evaporation using a rotary evaporator. Then, the product CTPP–PEG–OH was precipitated with ice ether and purified with the silica gel column (200–300 mesh). After that, 0.2 g CTPP–PEG–OH was dissolved in 0.8 mL dichloromethane containing 0.02 mL hydrochloric acid (HCl), 0.3 mL ether and 0.8 mL ε-caprolactone, reacting for 24 h at room temperature with the HCl and ether as catalyst. After the reaction, the product was precipitated with 30 mL ice ether, washed with water to remove unreacted substances, and then dried in vacuum. The obtained CTPP–PEG–PCL polymer was purified by the purification method similar to CTPP–PEG–OH and dried in vacuum for 48 h.

For structure confirmation, the synthesized CTPP–PEG–OH polymer was analyzed by Fourier transform infrared spectroscopy (FTIR) and ^1^H nuclear magnetic resonance (^1^H NMR). The CTPP–PEG–PCL polymer was demonstrated by ^1^H NMR.

### 2.5 Preparation of curcumin-loaded micelles

Cur-loaded micelles were prepared by a self-assembly solvent evaporation method as published literature.^40^ Briefly, Cur was mixed with CTPP–PEG–PCL or mPEG–PCL polymers with different weight ratio (1 : 17, 1 : 10, 1 : 7, 1 : 5) and the complexes was completely dissolved in acetone. After that, the organic solution was added into deionized water dropwise with stirring for at least 6 h under dark condition until the organic phase completely volatilized. The residual organic solvent was removed by vacuum drying, and then the unencapsulated curcumin was dialyzed by a dialysis bag (MW 4000) to obtain micelles loaded with curcumin. CTPP–PEG–PCL or mPEG–PCL micelles were prepared as control in the same way.

### 2.6 Characterization of Cur–CTPP–PEG–PCL micelles

#### 2.6.1 Hydrodynamic diameter and zeta potential

Marvin Nano ZS90 (Malvern, UK) was used to measure the particle size and zeta potential of Cur–CTPP–PEG–PCL micelles at a constant scattering angle of 90°. Atomic Force Microscopy (AFM) was used to observe the morphology of micelles. The samples in aqueous buffer were dispersed onto the surface of mica, dried with compressed air for 24 h. Then, samples were fulfilled by means of the tapping mode using an atomic force microscopy (Nano Scope IIIa, Veeco, USA).

#### 2.6.2 Critical micelle concentration

The critical micelle concentration (CMC) of blank CTPP–PEG–PCL micelles was measured using pyrene as a fluorescent probe. Various concentration of blank micelles (10^−5^, 10^−4^, 0.0004, 0.001, 0.004, 0.01, 0.04, 0.1, 0.4, 0.8 mg mL^−1^) was mixed with 1 μg mL^−1^ pyrene, after sonicated for 30 min and incubated for 1 h at 40 °C, mixtures were kept overnight in dark condition. The pyrene fluorescence of formulations was measured by a fluorescence spectrometer (Lumina, Thermo Fisher Scientific, MA, USA) with emission spectra from 350 nm to 450 nm and the excitation wavelength at 339 nm. The intensity ratio of pyrene fluorescence at 373 nm (*I*_373_) to 384 nm (*I*_384_), changing with the amount of pyrene entered into micelles from the aqueous phase, was calculated.

#### 2.6.3 Encapsulation efficiency (EE) and drug loading (DL) of micelles

The content of curcumin encapsulated in CTPP–PEG–PCL micelles was determined by ultraviolet spectrophotometry (UV) (ESI methods 1†). Briefly, in order to disrupt the polymer shells, micelles were completely dissolved in methanol and sonicated for 10 min. Then, after being filtered through 0.45 μm filter membrane, the obtained solution was centrifugated at 6000 g for 20 min by ultrafiltration tube (MW = 1000). The supernatant was measured at 426 nm by UV spectrophotometer. The standard curve of curcumin was established to calculate its concentration by UV. The encapsulation efficient and drug loading of micelles were calculated according to the following formula:
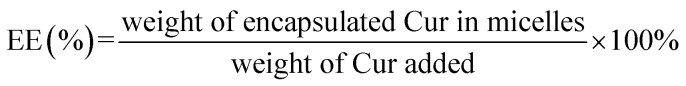

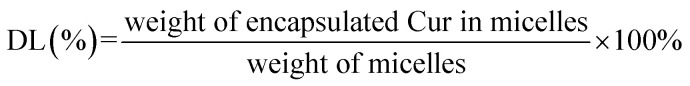


#### 2.6.4 *In* vitro release of curcumin

The membrane dialysis method was used for the drug release profile of CTPP–PEG–PCT. Cur, Cur–mPEG–PCL, and Cur–CTPP–PEG–PCT were added into a dialysis bag (MW 4 kDa). The end-sealed dialysis bag was placed in 30% ethanol in PBS (0.1 M, pH7.4) was used as dissolution medium at 37 °C, and medium was collected at determined time point. The amount of Cur released in the medium was monitored by UV-vis spectroscopy, and the experiments were repeated for three times.

#### 2.6.5 Stability of micelles

To inspect the physical stability of Cur-loaded CTPP–PEG–PCL micelles, DLS was adopted to measure particle size and *ζ* potential. Micelles were stored at 4 °C for four weeks. In addition, the stability of micelles in serum solution was measured by suspending with 10% FBS for 24 h at room temperature.

### 2.7 Cellular uptake study

To trace the cellular uptake of the micelles, HSC-T6 cells and AML 12 cells were seeded in 24-well plates at the density of 1 × 10^4^ cells per well and incubated overnight for cell attachment. After treated with Cur, Cur–mPEG–PCL, and Cur–CTPP–PEG–PCL micelles (100 μg mL^−1^ of Cur) for 1 h, 2 h, and 4 h, cellular uptake was terminated by cold PBS. Then, the cells were trypsinized, harvested and washed with PBS. The intracellular fluorescent intensity in HSC-T6 cells was determined by the flow cytometer (FACS, MACSQuant Analyzer 10, Miltenyi, Germany).

### 2.8 Cell viability assay

In order to investigate the biocompatibility of blank micelles, MTT assay was used to measure the cell viability. HSC-T6 cells and AML-12 cells were seeded in 96-well at the density of 5000 cells per well separately, and incubated at 37 °C under 5% CO_2_ overnight. Cells were incubated with various concentration of mPEG–PCL, CTPP–PEG–PCL micelles for 48 h. The medium was replaced with MTT solution (5 mg mL^−1^ in sterile PBS) and incubated for further 4 h. The supernatant was aspirated carefully and DMSO (150 μL) was added to dissolve the formed formazan crystals. Finally, the absorbance was detected at 490 nm by using a microplate reader (ELx800, BioTek Instruments, Winooski, VT) to measure the cell viability (%) of each group. The cytotoxicity of Cur and Cur-loaded micelles (Cur–mPEG–PCL, Cur–CTPP–PEG–PCL) with a serious range of concentration was evaluated by MTT assays. The operation was consistent with the above description.
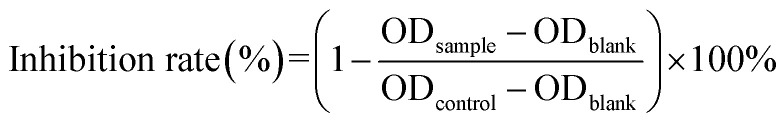


### 2.9 Endosomal escape

Lysosome escape is one of the necessary conditions for micelles nanoparticles to target mitochondria. To verify that Cur–CTPP–PEG–PCL micelles could escape from endosomes, Cur–CTPP–PEG–PCL micelles were incubated with HSC-T6 cells for 1, 3 and 5 h. The cells were washed with PBS and stained with LysoTracker Red as the probe of endosomes after incubation at each timepoint. HSC-T6 cells were fixed with 4% paraformaldehyde for10 min and subsequently treated with Hoechst 33258 for 10 min to stain the nuclei. Finally, the cells were observed under confocal laser scanning microscope (Leica, Germany) by using 63× magnification oil immersion lenses.

### 2.10 Co-localization study

In order to evaluate the targeting ability of micelles, confocal laser scanning fluorescence microscope (CLSM, Leica, Germany) was used to observe the co-localization of Cur and mitochondria. Briefly, exponentially growing HSC-T6 cells were seeded in a 15 mm^2^ dish at the density of 10^5^ cells per dish and incubated at 37 °C for 24 h in 5% CO_2_. After incubation, the cells were treated with free Cur, Cur–mPEG–PCL or Cur–CTPP–PEG–PCL, and then incubated at 37 °C for another 6 hours under 5% CO_2_ conditions. Then, the cells were washed with PBS, fixed with 4% paraformaldehyde for 10 min, and stained with mitochondrial fluorescent probe Mitotracker Red and nuclear fluorescent probe Hoechst 33258 for 10 min. Images could be observed by CLSM.

### 2.11 Measurement of mitochondria membrane potential (MMP)

Fluorescent probe JC-1 was used to monitor the mitochondria membrane potential. In short, HSC-T6 cells were seeded into 24-well plates at the density of 10^4^ cell per well and grown for 24 h at 37 °C for in 5% CO_2_. Then, cells were incubated with CTPP–PEG–PCL, mPEG–PCL, Cur, Cur–mPEG–PCL, Cur–CTPP–PEG–PCL for 48 h. After being washed with PBS three times, the cells were incubated with 10 mg mL^−1^ JC-1 for 30 min at 37 °C. Finally, the fluorescent intensity of green and red was measured by microplate reader (Bio-rad, USA). Hydroxycamptothecin was used as the positive control. MMP was calculated as the ratio of the red to green fluorescence intensity of JC-1 and the value was normalized by control group.

### 2.12 Hemolysis test

In order to evaluate the safety of micelles, the fresh mouse blood was centrifuged at 4000 rpm for 6 min, and the deposited red blood cells were washed with 0.9% NaCl solution after discarding the supernatant.^41^ Then, it was prepared into a 2% red blood cell suspension with 0.9% NaCl solution. After that, the suspension was incubated with different concentration gradients Cur, Cur–mPEG–PCL, Cur–CTPP–PEG–PCL micelles at 37 °C for 1 hour. The mixture was then centrifuged at 3000 rpm for 10 min. Finally, UV spectrophotometer was used to monitor the absorbance at 541 nm to investigate the hemolysis. Deionized water and 0.9% NaCl were used as the positive and negative control, respectively. The hemolysis ratio was calculated as following:
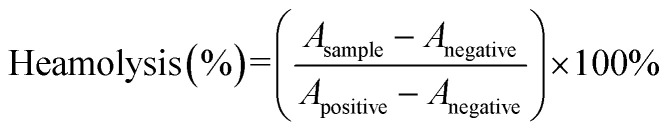


The criterion for hemolysis was that if the hemolysis ratio was less than 5%, it was considered non-toxic and safe for injection.

### 2.13 *In vivo* pharmacokinetics study

Pharmacokinetics were studied in SD rats. SD rats were randomly divided into three groups with each group of three rats. Then, Cur solution, Cur–mPEG–PCL, and Cur–CTPP–PEG–PCL micelles at an equivalent dose of 17 mg kg^−1^ Cur were administered through tail vein. Blood samples (0.5 mL) were extracted from the rat orbital sinus into heparinized centrifuge tubes at 3, 5, 10, 15, 30, 45, 60, 120 min and then centrifuged at 8000 rpm for 5 min to obtain the upper plasma. After that, Cur in plasma samples were extracted by adding ethyl acetate and vertexing for 5 min, then centrifuged at 5000 rpm for 5 min to get supernatant. Finally, the amount of Cur in the supernatant was measured by high-performance liquid chromatography (HPLC) based on established analytical method (SI). Various pharmacokinetic parameters (AUC, MRT, *t*_1/2_, *C*_max_, CL, and *V*) were calculated according to the analysis of DAS3.0 software.

### 2.14 *In vivo* pharmacodynamic experiment

The mice were divided into five groups with five in each group randomly. The liver fibrosis model was established by injecting carbon tetrachloride (CCl_4_, 40% in olive oil, 1 mL kg^−1^) intraperitoneally twice a week for four weeks. The mice were only administrated the same dosage of olive oil in control group. In order to investigate the regression of liver fibrosis, normal saline, Cur, Cur–mPEG–PCL, and Cur–CTPP–PEG–PCL micelles were injected through tail vein twice a week for eight weeks (Cur dosage: 17 mg kg^−1^). After treatment through veil tail, the mice were sacrificed and blood and livers were collected for the following study. After embedding in paraffin, the liver samples were cut into 5 μm slices. Then, liver slices were stained with hematoxylin–eosin staining (H&E) and Sirius Red staining solution for 2 h. After dehydration and fixation, the slices were observed and photographed by microscope.

### 2.15 Statistical analysis

All data in the manuscript were presented as mean ± SD. An unpaired two-tailed Student's *t*-test was used to compare the difference between two groups and one-way ANOVA was carried out for the analysis of multigroup comparison. *p < 0.05 indicated a significant difference.

## 3. Result and discussion

### 3.1 Characterization of CTPP–PEG–PCL copolymer

**Fig. 1:**
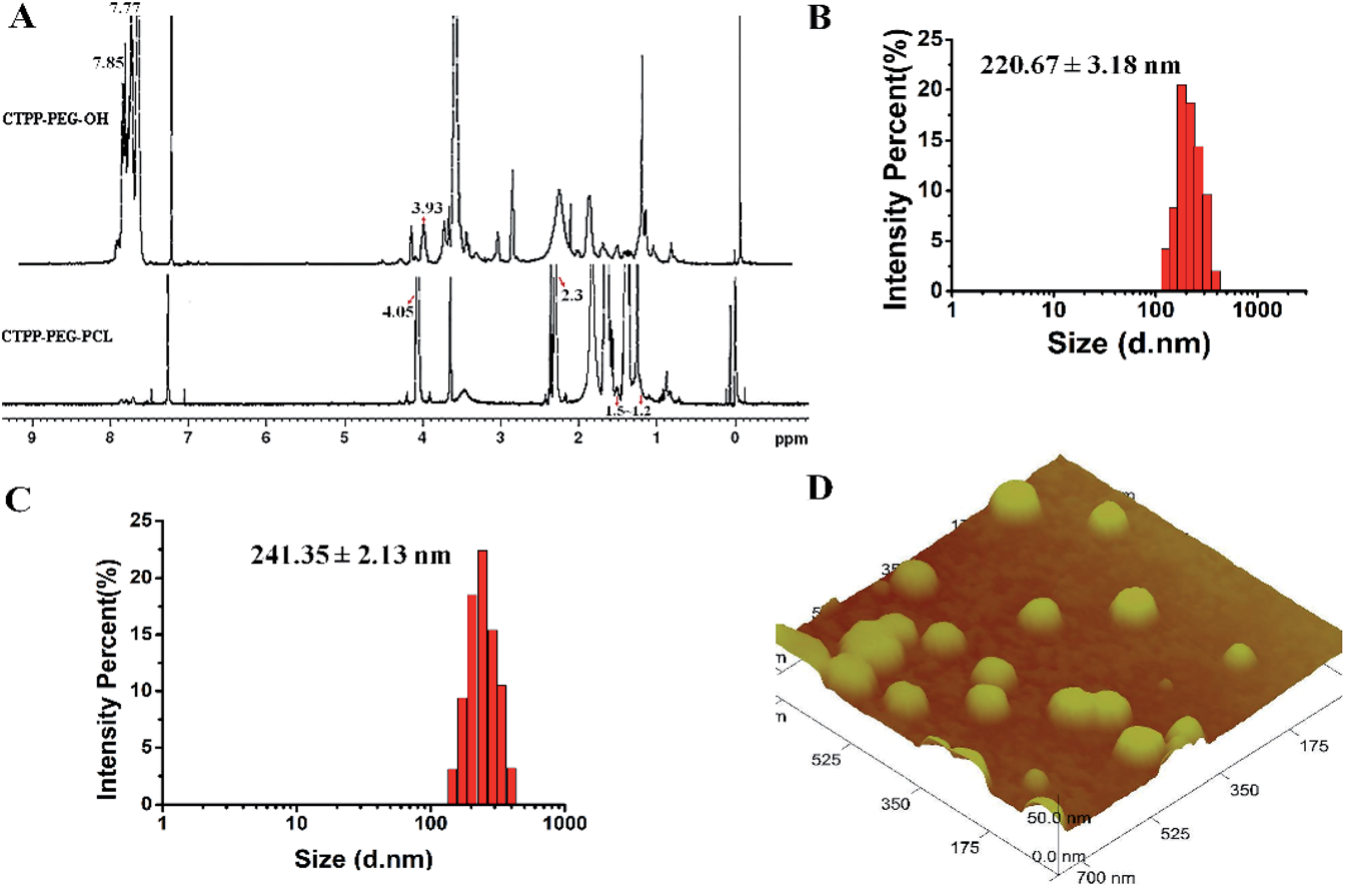
(A) The ^1^H NMR spectra of CTPP–PEG–OH and CTPP–PEG–PCL. (B) Particle distribution of mPEG–PCL micelles by DLS. (C) Particle distribution of CTPP–PEG–PCL micelles by DLS. (D) Image of CTPP–PEG–PCL micelles measured by atomic force microscopy (AFM).

The preparation process of polymer CTPP–PEG–PCL was expressed in Scheme 1. CTPP–PEG–OH was synthesized *via* esterification reaction of carboxylated CTPP and PEG using DCC as the condensation agent and DMAP as the catalyst. Then, the linear CTPP–PEG–PCL polymer was synthesized with the hydrochloric acid and ether as catalysts under room temperature. FTIR spectra and ^1^H NMR spectra were performed to confirm the successful conjugation of CTPP and PEG. The FTIR spectrum of CTPP–PEG–OH was illustrated in Fig. S1.† As shown in the spectrum of CTPP–PEG–OH, 1280.00 cm^−1^ belonged to –C–O–C, 1730.00 cm^−1^ belonged to C=O, 3417.72 cm^−1^ belongs to OH in –CH_2_–OH, 1600 cm^−1^, 740.84 cm^−1^, 842.28 cm^−1^, 691.78 cm^−1^ were all characteristic peaks of aromatics. The FTIR spectrum of CTPP–PEG–OH had characteristic peaks of PEG and CTPP, suggesting that the intermediate product CTPP–PEG–PCL was successfully synthesized. The ^1^H NMR spectra was detected to confirm the successful synthesis of CTPP–PEG–PCL. As shown in Fig. 1A, the typical peak of aromatic protons could be seen at 7.77–7.85 ppm in the CTPP–PEG–OH spectra, while the H signal of CH_2_ belong to PEG appeared at 3.93 ppm. In the ^1^H NMR spectra of CTPP–PEG–PCL, in addition to having the above proton characteristic peaks, the proton signal of CH_2_ belong to PCL could be observed at 1.2–1.5 ppm, 2.3 ppm and 4.05 ppm, proving the successful construction of CTPP–PEG–PCL. According to the analysis of ^1^H NMR, the percentage of CTPP successfully couple to PEG–PCL was 10.5%.

### 3.2 Particle size, zeta potential and morphology of CTPP–PEG–PCL micelles

The hydrodynamic diameter and zeta potential were detected by DLS. As shown in Table 1, Fig. 1B and C, the particle sizes of mPEG–PCL, CTPP–PEG–PCL, Cur–mPEG–PCL and Cur–CTPP–PEG–PCL were 198 ± 3 nm, 224 ± 3 nm, 221 ± 3 nm and 241 ± 2 nm. The functionalization of CTPP increased particle size of the formed micelles. Similarly, the diameter of Cur-loaded micelles increased with the exist of Cur. The PDI of micelles was all less than 0.3, indicating that the micelles prepared based on CTPP–PEG–PCL had excellent dispersion. The surface zeta potential of mPEG–PCL, CTPP–PEG–PCL, Cur–mPEG–PCL and Cur–CTPP–PEG–PCL were −20 ± 2 mV, −15 ± 3 mV, −19 ± 3 mV and −10 ± 3 mV, indicating excellent physical stability and less systemic toxicity. The zeta potential of Cur–CTPP–PEG–PCL was larger than Cur–mPEG–PCL, which might be due to the positive charge of CTPP.

**Table 1:** Particle size, PDI, and zeta potential of CTPP–PEG–PCL micelles (*n* = 3)

Micelles	Particle size (nm)	Polydispersity	Zeta potential (mV)
mPEG–PCL	198 ± 3	0.19 ± 0.07	−20 ± 2
CTPP–PEG–PCL	224 ± 3	0.25 ± 0.10	−15 ± 3
Cur–mPEG–PCL	221 ± 3	0.21 ± 0.10	−19 ± 3
Cur–CTPP–PEG–PCL	241 ± 2	0.18 ± 0.01	−10 ± 3

Furthermore, AFM image was conducted to observe the morphology of CTPP–PEG–PCL micelles. As shown in Fig. 1D, the average particle size of the micelles was 98 ± 2 nm, which was less than that detected by DLS. Meanwhile, the micelles had a spherical shape with smooth surface and well dispersed. This might be caused by shrinkage after drying, which was commonly reported in literature.

### 3.3 Critical micelle concentration (CMC)

**Fig. 2:**
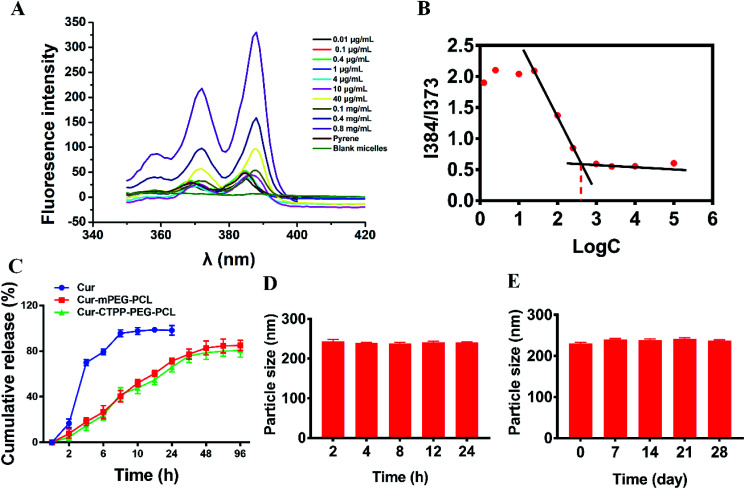
(A) Fluorescence excitation spectra of pyrene with different concentration micelles solution. (B) Critical micelle concentration of CTPP–PEG–PCL micelles detected by pyrene according to section A. (C) *In vitro* release profiles of Cur, Cur–mPEG–PCL and Cur–CTPP–PEG–PCL micelles (*n* = 3). (D) Stability of the Cur–CTPP–PEG–PCL micelle in PBS containing 10% FBS within 24 h. (E) Stability of the Cur–CTPP–PEG–PCL micelle after stored at 4 °C for four weeks.

CMC is the important parameter reflecting the formation concentration and stability of micelles.^42^ Pyrene was used as a fluorescent probe to determine the CMC of CTPP–PEG–PCL micelles. As shown in Fig. 2A and B, the fluorescence intensity of pyrene changed with the concentration of the CTPP–PEG–PCL copolymer. The fluorescence intensity of pyrene was increased with the concentration of CTPP–PEG–PCL increasing, and it increased sharply when micelles were formed. The CMC of CTPP–PEG–PCL micelle was 0.002620 mg mL^−1^, indicating that micelles had high stability and ability to maintain their structure under dilution conditions.

### 3.4 Drug loading and *in vitro* release of Cur

The UV spectrophotometric method illustrated in ESI† method 2 was established to determine the content of Cur in micelles. The Cur concentration was used as an abscissa and the absorbance was used as the ordinate for standard curve construction: *y* = 135782*x* + 0.0054 (*R*^2^ = 0.9997). As displayed in Table 2, different ratio of Cur to CTPP–PEG–PCL was screened. As the ratio of CTPP–PEG–PCL polymer increasing, the drug loading increased. However, the encapsulation efficiency increased first and then decreased, exhibiting highest at the ratio of 1 : 10. The drug loading and encapsulation efficiency of Cur–CTPP–PEG–PCL was 6% ± 0 and 93% ± 2, respectively. Hence, the ratio of 1 : 10 was selected for future follow-up research.

**Table 2:** Drug loading and encapsulation efficiency of Cur–CTPP–PEG–PCL micelles (*n* = 3)

Ratio of Cur : CTPP–PEG–PCL	Drug loading (%)	Encapsulation efficiency (%)
1 : 17	5 ± 1	74 ± 2
1 : 10	6 ± 0	93 ± 2
1 : 7	8 ± 0	89 ± 2
1 : 5	13 ± 1	26 ± 2

The controlled release of micelles in the targeting site is one of the key performance indexes of nanoparticles in treatment. The *in vitro* drug release curves of micelles were shown in Fig. 2C. Free Cur was released quickly, while Cur in CTPP–PEG–PCL and mPEG–PCL micelles tended to be released slowly, showing better sustained-release properties. The excellent sustained-release behavior of Cur loaded CTPP–PEG–PCL micelles might increase the bioavailability of Cur *in vivo*.

### 3.5 Stability analysis of Cur–CTPP–PEG–PCL micelles

The stability of Cur–CTPP–PEG–PCL micelles in 10% FBS and stored in 4 °C was measured by DLS. Results in Fig. 2D illustrated that the particle size of Cur–CTPP–PEG–PCL micelles had no significant change in the presence of 10% FBS. Similarly, particle size remained unchanged compared with fresh prepared after stored at 4 °C for four weeks (Fig. 2E). Altogether, Cur–CTPP–PEG–PCL micelles had better stability at stored conditions and physiological condition without any aggregate.

### 3.6 Cytotoxicity analysis

**Fig. 3:**
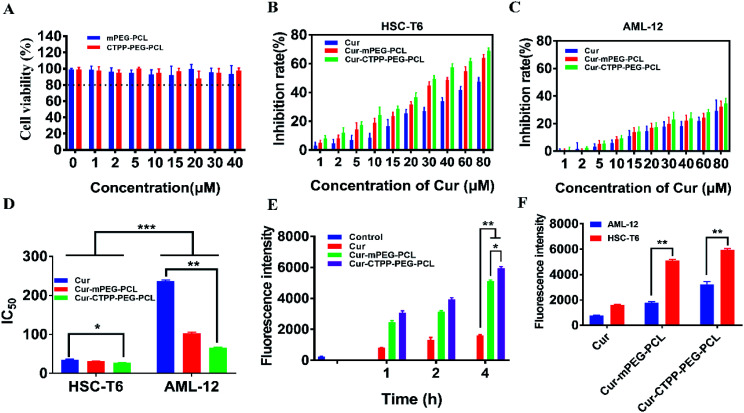
(A) *In vitro* cell viability of HSC-T6 cells after treated with mPEG–PCL and CTPP–PEG–PCL micelles at different concentration (*n* = 3). *In vitro* cell inhibition of Cur, Cur–mPEG–PCL and Cur–CTPP–PEG–PCL micelles against HSC-T6 cells (B) and AML-12 cells (C) at different concentration (*n* = 3). (D) IC_50_ values of Cur, Cur–mPEG–PCL and Cur–CTPP–PEG–PCL micelles on HSC-T6 cells and AML-12 cells. (E) *In vitro* cellular uptake studies of Cur, Cur–mPEG–PCL and Cur–CTPP–PEG–PCL micelles by flow cytometry in HSC-T6 cells. (F) The *in vitro* cellular uptake studies of Cur, Cur–mPEG–PCL and Cur–CTPP–PEG–PCL micelles by flow cytometry in AML-12 cells and HSC-T6 cells at 4 h. *p < 0.05, **p < 0.01, ***p < 0.001.

MTT assay was used to evaluate the biocompatibility of mPEG–PCL and CTPP–PEG–PCL blank micelles on HSC-T6 cells and AML-12 cells. As shown in Fig. 3A, cell viability of HSC-T6 cells after treatment with mPEG–PCL and CTPP–PEG–PCL at the concentration range from 1–40 μM was all above 80%. Notably, blank micelles displayed excellent biocompatibility.

After that, we evaluated the potential cytotoxicity of Cur–CTPP–PEG–PCL micelles on HSC-T6 cells for 48 h. As shown in Fig. 3B, the cell viability of HSC-T6 treated with Cur, Cur–mPEG–PCL and Cur–CTPP–PEG–PCL micelles was significantly inhibited in a concentration-dependent manner. A lowest cell inhibition rate was exhibited in free Cur group, indicating that it was difficult to exert therapeutic effects for free Cur. In compared with free Cur, the inhibition rate showed enhanced cellular efficacy after incubated with Cur–mPEG–PCL micelles. With the assistance of mPEG–PCL vehicle, more Cur could be delivered into the cell and exhibited the cytotoxicity. In addition, Cur–CTPP–PEG–PCL micelles showed the most effective inhibition effect, and the IC_50_ value showed a significantly difference with that of the free Cur group (Fig. 3D). Moreover, the IC_50_ of Cur–CTPP–PEG–PCL group was lower than Cur–mPEG–PCL group, suggesting that the presence of CTPP might enable Cur–CTPP–PEG–PCL to targeting mitochondrial, thereby resulting in enhanced cell inhibition. In addition, we also investigated the cytotoxicity of different formulations on AML-12 cells for 48 h. As presented in Fig. 3C, all formulations exhibited lower cell inhibitory efficiency in AML-12 cells, and the IC_50_ exhibited a significant difference with that of HSC-T6 cells. As reported by Emestina Marianna De Francesco *et al.*,^43^ at concentrations of CTPP toxic only for cancer cells, but not for normal cells. Therefore, we trusted that the designed mitochondrial-targeted nanoparticles may be a potential vehicle for anti-fibrosis treatment.

### 3.7 *In vitro* cellular uptake

Effective cellular uptake is essential for the Cur to generate cellular therapeutic effects. To investigate the cellular delivery of Cur mediated by CTPP micelles, we measured the fluorescence intensity of Cur in HSC-T6 cells by flow cytometry. As shown in Fig. 3E, the cellular uptake of Cur–mPEG–PCL and Cur–CTPP–PEG–PCL micelles increased with the incubation time extended from 1 h to 4 h, displaying a time-dependent manner. The accumulation of free Cur in HSC-T6 cells was lowest and had significantly difference with that of Cur-loaded micelles, indicating that the existence of micelles could effectively increase the cellular uptake ability of Cur. Compared with the Cur group, the cellular uptake of mPEG–PCL micelles increased, showing a significant difference (*p* < 0.05), which might depend on the presence of PEG. The previous research demonstrated that mPEG could gift the nanoparticles of selective organ targeting ability, thereby increasing the cellular uptake.^44^ Cur–CTPP–PEG–PCL micelles exhibited a significant difference in HSC-T6 cells accumulation compared with Cur–mPEG–PCL. The enhanced cellular uptake might be caused by the positively charged CTPP. Meanwhile, AML-12 cells were selected as a control for the *in vitro* cellular uptake. We measured the fluorescence intensity of Cur in AML-12 cells by flow cytometry after incubation with micelles for 4 h. The accumulation of Cur in AML-12 cells was enhanced with the assistant of mPEG–PCL and CTPP–PEG–PCL micelles (Fig. 3F). Nevertheless, the fluorescence intensity of all formulations in AML-12 cells showed extremely significant differences with that in HSC-T6 cells. This might indicate that micelles were more likely to uptake by diseased cells, thereby reducing the side effects on normal cells. The reduced cellular intake might harbinger the low cytotoxicity and poor efficacy.

### 3.8 Endosomal escape

**Fig. 4:**
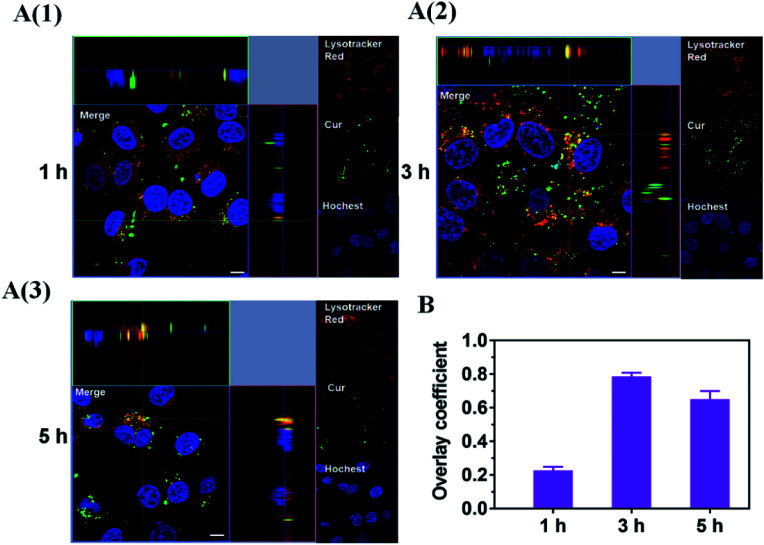
(A) Endosome escape of Cur–CTPP–PEG–PCL micelles after incubate with HSC-T6 cells for 1, 3 and 5 h. Endosome was indicated by Lyso-Tracker Red. Nucleus was stained by Hoechst 33 258 (blue). Scale bar, 10 μm. Each picture shows a larger top down *xy*-view, the horizontal and the vertical lines show the position within the image of the *xz*- and *yz*-projections given in the lower and right panels respectively, allowing to identify co-localization of Cur and endosome. (B) The overlay coefficient of section A quantized by Image J.

Considering the endosomal escape was a necessary condition to enter mitochondria, we investigated the endosomal escape of Cur–CTPP–PEG–PCL micelles. As presented in Fig. 4A and B, the green fluorescence of Cur was mainly overlapped with the red endosomal fluorescence to generate yellow fluorescence after 3 h incubation with HSC-T6 cells. With the time prolonged, the dissociation of green and red was observed, indicating the Cur-loaded micelles could escape from the endosomes successfully. The overlay coefficient was analyzed by Image J according to the Fig. 4A, which presented the highest in 3 h while decreased in 5 h, suggesting that micelles had the ability to escape from endosomes. The endosomal escape ability might be attributed to the positively charge of CTPP. The CTPP–PEG–PCL micelle successfully escaped from the lysosome, suggesting that it might have the potential to target mitochondria.

### 3.9 Co-localization of Cur–CTPP–PEG–PCL and mitochondria

**Fig. 5:**
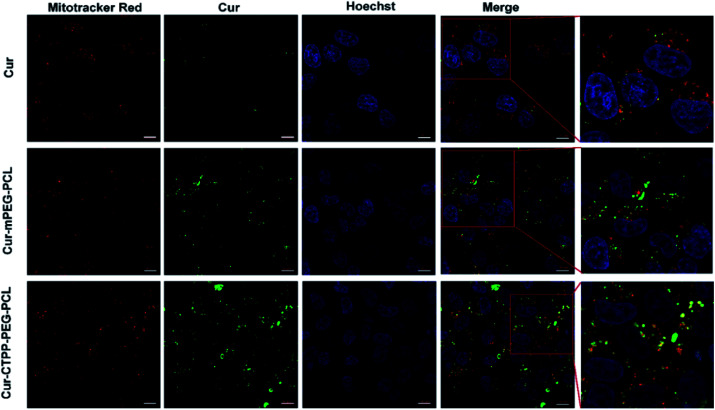
Mitochondrial targeting effect ofc Cur–CTPP–PEG–PCL micelles after incubation with HSC-T6 cells for 6 h. Mitochondrial was indicated using Mitotracker Red (red). The nucleus was stained with Hoechst 33258 (blue). The green fluorescence indicated the Cur. The scale bar was 10 μm.

CLSM was used to display the subcellular distribution of Cur-loaded micelles in HSC-T6 cells after co-incubation for 6 h. As shown in Fig. 5, red fluorescence represented mitochondria and green fluorescence represented Cur. The yellow fluorescence overlapped of red and green fluorescence indicated the co-localization of Mitotracker Red and Cur. In the cells treated with CTPP–PEG–PCL micelles, a large number of yellow fluorescence dots were observed, while some yellow fluorescence dots were observed along with the green fluorescence dot distribution after treated with mPEG–PCL micelles. This indicated that CTPP with the mitochondrial targeting effect allowed Cur selectively aggregate in mitochondria. However, after treated with free Cur, only weak green fluorescence and a small amount of yellow fluorescence were observed in HSC-T6 cells, indicating that it was difficult for free Cur to enter cells and mitochondria. Altogether, the co-localization results suggested that CTPP had a targeting ability to mitochondria and could effectively deliver Cur to the mitochondria of cells.

### 3.10 Detection of MMP

**Fig. 6:**
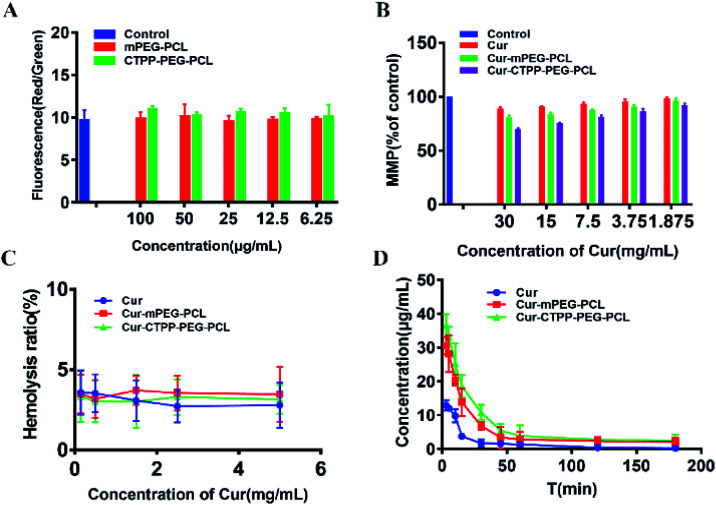
(A) MMP of blank mPEG–PCL and CTPP–PEG–PCL micelles detected by staining with JC-1 dye. (B) MMP of Cur and Cur-loaded micelles detected by staining with JC-1 dye. (C) Hemolysis analyses of Cur, Cur–mPEG–PCL and Cur–CTPP–PEG–PCL micelles *in vitro*. (*n* = 3) (D) Pharmacokinetic parameters of Cur, Cur–mPEG–PCL and Cur–CTPP–PEG–PCL micelles in mice after injecting through tail vein (*n* = 4).

Apoptosis is mainly triggered by internal and external pathways. The intrinsic apoptotic pathway is activated in response to cell damage, such as DNA damage and oxidative stress damage, which leads to permeability of the mitochondrial membrane and release of proteins from the mitochondrial membrane space.^45^ Since the damage of the mitochondrial membrane occurred in the early stage of apoptosis, JC-1 was used as MMP-sensing probe to detect the level of MMP. As shown in Fig. 6A, the MMP of blank micelles at different concentration range exhibited no change in MMP compared with Control, suggesting that the CTPP–PEG–PCL and mPEG–PCL polymers could not affect the MMP. After treated with Cur, Cur–mPEG–PCL and Cur–CTPP–PEG–PCL micelles for 24 h, a loss in MPP could be observed with a concentration-dependent manner (Fig. 6B). The mitochondrial membrane potential of Cur–CTPP–PEG–PCL micelles was significantly different from that of Cur–mPEG–PCL and free Cur, indicating that the presence of CTPP could targeted to the mitochondria and destroyed mitochondria. The reduction of MMP was consistent with the results of mitochondrial targeting. Micelles was targeted into mitochondria due to the present of CTPP, leading to the changes in membrane potential. At the same time, the cell status was observed under a microscope. The cell inhibition effect of CTPP–PEG–PCL micelles was highest, which was consistent with the above results of cytotoxicity, indicating that the reduction of membrane potential could inhibit the cell proliferation.

### 3.11 Hemolysis test

In order to investigate the injection safety of Cur, Cur–mPEG–PCL, and Cur–CTPP–PEG–PCL micelles, the hemolysis test of micelles was measured. As displayed in Fig. 6C, the hemolysis ratio was all less than 5% under different concentration range from 0.15–5 mg mL^−1^, indicating that CTPP–PEG–PCL based micelle formulation had a better biocompatibility with red blood cells and biocompatibility for *in vivo* administration. However, the blood is composed of a various of cell types (such as immune related cells). The interaction of Cur-loaded micelles with these cells will cause the limited Cur reaching the diseased site, so it is necessary to reduce the interaction of micelles with other cells in the blood. As reported by Mai N. Vu *et al.*,^46^ small and negatively charged nanoparticles seem to be the promising candidates for the cancer therapeutic drugs due to their low cellular interaction with immune cells.

### 3.12 Pharmacokinetic experiment

The pharmacokinetic of Cur relative formulations was investigated in mice by intravenous injection. The content of Cur in plasma was detected by HPLC. The average plasma concentration–time curves of Cur, Cur–mPEG–PCL, and Cur–CTPP–PEG–PCL micelles were displayed in Fig. 6D, and the corresponding pharmacokinetic parameters were model fitted by DAS 3.0 software in Table 3. The blood elimination half-life of Cur, Cur–mPEG–PCL and Cur–CTPP–PEG–PCL micelles was 48.12 ± 14.83 min, 107.43 ± 15.43 min and 114.88 ± 10.51 min, respectively. Compared with the Cur group, the plasma concentration of Cur in mPEG–PCL and CTPP–PEG–PCL micelles decreased more slowly and had obviously sustained release characteristics, which was consistent with the sustained release performance of the delivery system *in vitro* release results. Obviously, the area under the plasma concentration curves (AUC) of Cur–mPEG–PCL and Cur–CTPP–PEG–PCL micelles were much higher than free Cur, illustrating the better bioavailability of micelles formulations. The expected bioavailability improvement of Cur–mPEG–PCL and Cur–CTPP–PEG–PCL micelles in pharmacokinetics might be attributed to the sustained release delivery system, prolonging the long circulation of Cur *in vivo*.

**Table 3:** Pharmacokinetic parameters of formulations after intravenous administration (*n* = 3)

Parameters	Cur	Cur–mPEG–PCL	Cur–CTPP–PEG–PCL
AUC_0–*t*_ (μg mL^−1^ min^−1^)	316.88 ± 20.03	705.67 ± 35.23	785.84 ± 19.21
AUC_0–∞_ (μg mL^−1^ min^−1^)	336.31 ± 36.41	968.38 ± 49.53	1052.43 ± 58.05
*C*_max_ (μg mL^−1^)	12.93 ± 1.51	30.54 ± 2.58	36.58 ± 3.28
*t*_1/2_ (min)	48.12 ± 14.83	107.43 ± 15.43	114.88 ± 10.51
AUMC (μg mL^−1^ min^−2^)	15 805.16 ± 7003.72	12 068.32 ± 2343.12	127 751.75 ± 19 225.11
MRT (min)	45.90 ± 14.55	112.34 ± 10.32	120.98 ± 11.31
CL (mg·(μg mL^−1^)^−1^·min^−1^)	0.05 ± 0.006	0.0231 ± 0.0018	0.0161 ± 0.0008

### 3.13 *In vivo* pharmacodynamic experiment

**Fig. 7:**
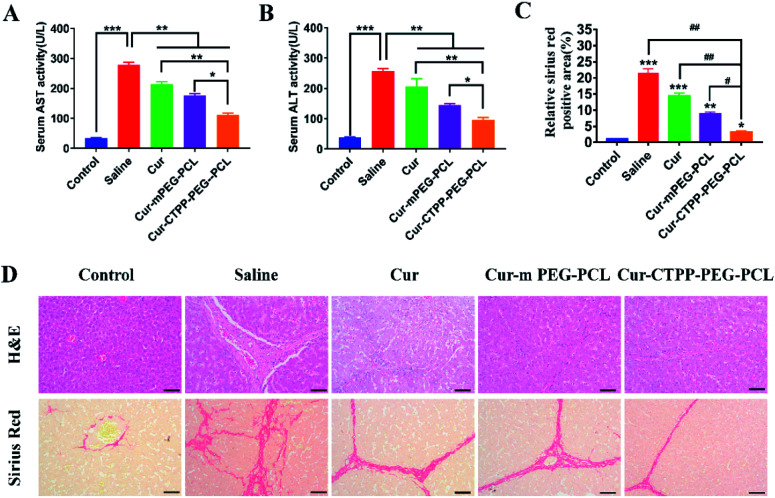
(A) The serum AST levels in mice blood; (B) the serum ALT levels in mice blood; (C) the semi-quantitative analysis of the Sirius red staining sections by Image J according to section D; (D) representative images of H&E staining and sirius red staining of livers after treatment with saline, Cur, Cur–mPEG–PCL, Cur–CTPP–PEG–PCL. (Scale bar: 100 μm) ***p < 0.001, **p < 0.01, *p < 0.05, ^###^*p* < 0.001, ^##^*p* < 0.01, ^#^*p* < 0.05.

To investigate whether Cur–CTPP–PEG–PCL micelles could improve the liver fibrosis, liver fibrosis model was established in mice by intraperitoneal injection of CCl_4_.^2^ The effect of Cur–CTPP–PEG–PCL micelles on liver fibrosis was evaluated by measuring biochemical indicators and H&E staining of liver tissue. The serum levels of AST and ALT in Cur, Cur–mPEG–PCL, and Cur–CTPP–PEG–PCL micelles were determined because the abnormal of AST and ALT in serum could reflect the liver damage degree indirectly. As displayed in Fig. 7A and B, the ALT and AST serum levels of the saline group injected with CCl_4_ were significantly higher than the normal group, indicating that the liver fibrosis model was constructed successfully. After treatment with Cur, Cur–mPEG–PCL, Cur–CTPP–PEG–PCL micelles, the levels of AST and ALT decreased significantly and had a significant difference with the saline group, illustrating that Cur-load micelles might be improve the liver fibrosis. Moreover, the decrease was most significant in the Cur–CTPP–PEG–PCL micelles treatment group, showing significant difference with Cur and mPEG–PCL. The enhanced improvement on liver fibrosis might be attributed to the mitochondrial targeting of CTPP. Moreover, the same results could be obtained in liver pathological slices of mice stained with H&E and Sirius red staining solution. As shown in Fig. 7D, the structure of live lobules was normal with little collagen deposition in control group, while abundant collagen fiber hyperplasia, pseudo-lobules, and severe hepatic lobule necrosis with lymphocyte infiltration could be observed in saline group. Compared with the saline group, the treatment of Cur, Cur–mPEG–PCL and Cur–CTPP–PEG–PCL micelles inhibited the liver fibrosis significantly. The degree of liver fibrosis was measured by Sirius Red staining using Image J analysis. As displayed in Fig. 7C, the semi-quantitative fibrosis area in livers of mice treated with Saline, Cur, Cur–mPEG–PCL and Cur–CTPP–PEG–PCL micelles covered the liver about 21.5%,14.4%, 8.9% and 3.2%. Compared with the model group (saline), the relative Sirius red positive area in all administration groups decreased, indicating that Cur had a certain therapeutic effect on liver fibrosis. Moreover, Cur–CTPP–PEG–PCL micelles had the lowest relative positive area of liver fibrosis, which was significantly different from Cur–mPEG–PCL micelles, suggesting that CTPP–PEG–PCL micelles could effectively deliver Cur to the mitochondrial action site. The enhanced anti-fibrosis effect might be due to the mitochondrial targeting of CTPP, leading to the apoptosis of fiber cells. In conclusion, Cur-loaded micelles delivery system functionalized with CTPP–PEG–PCL complex could effectively improve liver fibrosis.

## 4. Conclusion

Altogether, a micelle system based on CTPP–PEG–PCL complex with mitochondria-targeting was constructed to deliver the curcumin to the mitochondria of active HSC-T6 cells and prolong the long circulation and bioavailability of curcumin *in vivo* for the therapy of liver fibrosis. Cur-loaded micelles demonstrated the advantages of excellent encapsulation ability, low CMC and dilution stability. Compared with free Cur, Cur–CTPP–PEG–PCL micelles exhibited enhanced cellular uptake and cell proliferation inhibition in HSC-T6 cells. In addition, the modification of CTPP endowed micelles the endosomal escape ability and mitochondrial targeting ability. The targeting of mitochondria leaded to changes in mitochondrial membrane potential, and the results were consistent with that of cytotoxicity. Results of *in vitro* hemolysis test demonstrated that Cur-loaded micelles could be injected intravenously safely *in vivo*. Moreover, results in pharmacokinetic studies suggested that Cur–CTPP–PEG–PCL micelles could significantly enhance the bioavailability of Cur and extend the retention time of Cur *in vivo*. Furthermore, superior anti-liver fibrosis effect was exhibited after treated with Cur–CTPP–PEG–PCL micelles in mice with liver fibrosis. Altogether, the above results indicated that Cur–CTPP–PEG–PCL based micelles can serve as an effective targeted drug delivery system intracellularly, and can be a potential candidate for precision treatment of liver fibrosis in future clinical applications.

## Conflicts of interest

The authors report no conflicts of interest in this work.

## Supplementary Material

RA-011-D0RA09589C-s001

## References

[cit1] Parola M., Pinzani M. (2019). Mol. Aspects Med..

[cit2] Wang J. Z., Ding Y., Zhou W. (2020). Int. J. Pharm..

[cit3] Kitano M., Bloomston P. M. (2016). J. Clin. Med..

[cit4] Lee Y. A., Wallace M. C., Friedman S. L. (2015). Gut.

[cit5] Kisseleva T., Brenner D. A. (2007). J. Gastroenterol. Hepatol..

[cit6] Mann D. A., Smart D. E. (2002). Gut.

[cit7] Parola M., Pinzani M. (2019). Mol. Aspects Med..

[cit8] Tsuchida T., Friedman S. L. (2017). Nat. Rev. Gastroenterol. Hepatol..

[cit9] Schon H. T., Bartneck M., Kamphorst E. B., Nattermann J., Lammers T., Tacke F., Weiskirchen R. (2016). Front. Pharmacol..

[cit10] Bataller R., Brenner D. A. (2001). Semin. Liver Dis..

[cit11] Higashi T., Friedman S. L., Hoshida Y. (2017). Adv. Drug Delivery Rev..

[cit12] Yin C. Y., Evason K. J., Asahina K. J., Stainier D. Y. R. (2013). J. Clin. Invest..

[cit13] Goel A., Kunnumakkara A. B., Aggarwal B. B. (2008). Biochem. Pharmacol.

[cit14] Lin J. K. (2007). Adv. Exp. Med. Biol..

[cit15] Hong J. Y., Liu Y. Y., Xiao Y., Yang X. F., Su W. J., Zhang M. Z., Liao Y. H., Kuang H. X., Wang X. T. (2017). Drug Delivery.

[cit16] Zheng S. Z., Yumei F., Chen A. P. (2007). Free Radical Biol. Med..

[cit17] Lian N. Q., Jin H. H., Zhang F., Wu L., Shao J. J., Lu Y., Zheng S. Z. (2016). IUBMB Life.

[cit18] Lian N. Q., Jiang Y. Y., Zhang F., Jin H. H., Lu C. F., Wu X. F., Liu Y., Zheng S. Z. (2015). Lab. Invest..

[cit19] Zhang F., Lu C. F., Xu W. X., Shao J. J., Wu L., Lu Y., Zheng S. Z. (2016). J. Surg. Res..

[cit20] Zheng S. Z., Chen A. P. (2004). Biochem. J..

[cit21] Park E. J., Jeon C. H., Ko G., Kim J., Sohn D. H. (2000). J. Pharm. Pharmacol..

[cit22] Bruck R., Ashkenazi M., Weiss S., Goldiner I., Shapiro H., Aeed H., Genina O., Helpern Z., Pines M. (2007). Liver Int..

[cit23] Sela G. B., Epelbaum R., Schaffer M. (2010). Curr. Med. Chem..

[cit24] Guerrero S., Riquelme M. I., Orellana P. C., Garcia V. D., Lara P., Palma A. V., Cárdenas A., Miranda V., Robert P., Leyton L., Kogan M. J., Quest A. F. G., Ampuero F. O. (2018). Nanoscale.

[cit25] Altamimi M. A., Kazi M., Albgomi M. H., Ahad A., Raish M. (2019). Drug Dev. Ind. Pharm..

[cit26] Baghdan E., Duse L., Schüer J. J., Pinnapireddy S. R., Pourasghar M., Schäfer J., Schneider M., Bakowsky U. (2019). Eur. J. Pharm. Sci..

[cit27] Duse L., Agel M. R., Pinnapireddy S. R., Schäfer J., Selo M. A., Ehrhardt C., Bakowsky U. (2019). Pharmaceutics.

[cit28] Zhang M. M., Zhuang B., Du G. J., Han G., Jin Y. G. (2019). J. Pharm. Pharmacol..

[cit29] McBride H. M., Neuspiel M., Wasiak S. (2006). Curr. Biol..

[cit30] Green D. R., Reed J. C. (1998). Science.

[cit31] Yang Y. H., Karakhanova S., Hartwig W., D'Haese J. G., Philippov P. P., Werner J., Bazhin A. V. (2016). J. Cell. Physiol..

[cit32] Everett H., McFadden G. (2001). Virology.

[cit33] Mou S. F., Zhou Z. X., He Y. K., Liu F. X., Gong L. L. (2017). Oncol. Lett..

[cit34] Zhao L. L., Gu Q. R., Xiang L. T., Dong X. D., Li H. M., Ni J. Y., Wan L., Cai G. P., Chen G. R. (2017). Ther. Clin. Risk Manage..

[cit35] Yamada Y., Akita H., Kogure K., Kamiya H., Harashima H. (2007). Mitochondrion.

[cit36] Biswas S., Dodwadkar N. S., Piroyan A., Torchilin V. P. (2012). Biomaterials.

[cit37] Xiang H. J., Xue F. F., Yi T., Tham H. P., Liu J. G., Zhao Y. L. (2018). ACS Appl. Mater. Interfaces.

[cit38] Grossen P., Witzigmann D., Sieber S., Huwyler J. (2017). J. Controlled Release.

[cit39] Ma B. B., Sheng J., Wang P., Jiang Z. Y., Borrathybay E. (2019). Int. J. Nanomed..

[cit40] Xu Y. Q., Wang S. P., Chan H. F., Liu Y. L., Li H., He C. W., Li Z. Y., Chen M. W. (2017). Int. J. Pharm..

[cit41] Jin M. J., Piao S. J., Jin T. X., Jin Z. H., Yin X. Z., Gao Z. G. (2014). J. Huazhong Univ. Sci. Technol., Med. Sci..

[cit42] Liu J. Y., Pang Y., Huang W., Zhu X. Y., Zhou Y. F., Yan D. Y. (2010). Biomaterials.

[cit43] Francesco E. M. D., Ózsvári B., Sotgia F., Lisanti M. P. (2019). Front. Oncol..

[cit44] Zukancic D., Suys E. J. A., Pilkington E. H., Algarni A., Al-Wassiti H., Truong N. P. (2020). Pharmaceutics.

[cit45] Green D. R., Kroemer G. (2004). Science.

[cit46] Vu M. N., Kelly H. G., Wheatley A. K., Peng S., Pilkington E. H., Veldhuis N. A., Davis T. P., Kent S. J., Truong N. P. (2020). Small.

