# Supplementary material for: Mitochondria-targeted curcumin loaded CTPP–PEG–PCL self-assembled micelles for improving liver fibrosis therapy
Source: RSC Adv. 2021 Jan 28;11(10):5348–60. doi: 10.1039/d0ra09589c (PMC8694685; doi:10.1039/d0ra09589c)
Supplement: RA-011-D0RA09589C-s001 [file RA-011-D0RA09589C-s001.pdf]

Please do not adjust margins

Mitochondria-targeted curcumin loaded CTPP-PEG-PCL self-assembled micelles for improving liver fibrosis therapy

Received 00th January 20xx,
Accepted 00th January 20xx

DOI: 10.1039/x0xx00000x

Liqiao Zhang[a], Xiuhua Pan[d], Lixing Xu[b], Linlin Zhang[c*], Haiqin Huang[b*]

Methods

1. Quantitative measurement of the Cur loaded in CTPP-PEG-PCL micelles through UV-vis method

Accurately weigh 10.0 mg curcumin (Cur) into a 50 mL volumetric flask, add methanol to the volumetric flask and shake well to obtain a 200 μg/mL Cur standard solution. Dilute the Cur standard solution with methanol to different concentrations (1.6 μg/mL, 2.4 μg/mL, 3.2 μg/mL,4.0 μg/mL, 4.8 μg/mL, and 5.6 μg/mL). The absorbance (A) of different samples at 424 nm wavelength was measured by UV-vis spectrophotometer with methanol as blank control. The obtain standard curve is y=135782x+0.0054 (R^2=0.9997) (y: the absorbance value at 424 nm; x: concentration of Cur).

2. Quantitative measurement of the Cur *in vivo* by a high-performance liquid chromatography (HPLC) method

Cur content *in vivo* were determined by HPLC (Waters, USA) with the following conditions: an ThermoHypersil-keystone C18 column (4.6 mm × 250 mm, 5.0 μm); mobile phase: acetonitrile: 10% glacial acetic acid solution = 1:1 (v/v); detection wavelength 424 nm; flow rate 1.0 mL/min; column temperature room temperature; and injection volume 20 μL. Accurately weigh Cur 5.0 mg into a 50 mL volumetric flask, adding methanol and obtain 100 μg/mL Cur standard solution. After that, dilute to different concentration (0.1, 0.5, 2, 4, 10, 25 μg/mL) using 80% methanol-blank plasma mixed solution, and HPLC obtained the peak area of different samples. The peak area y was plotted on the ordinate, and the concentration x was plotted on the abscissa. The obtain standard curve is y=9.6321x + 10.361, R^2=0.9944 (y: peak area value at 424 nm; x: concentration of Cur).

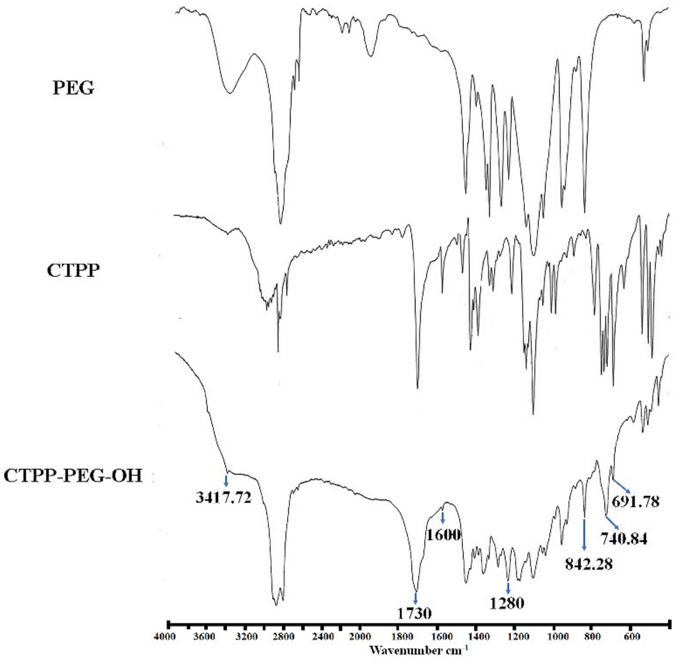

Figure S1. FIRT spectrum of PEG, CTPP and CTPP-PEG-OH.

[a] Department of Pharmacy, Chendu Women's and Children's Central Hospital, School of Medicine, University of Electronic Science and Technology of China, Chengdu 611731, PR China.
[b] College of Pharmacy, Nantong University, Nantong 226001, PR China.
[c] Nanjing Chia Tai Tianqing Pharmaceutical CO., Ltd, Nanjing 210000, PR China.
[d] Key Laboratory of Modern Chinese Medicines, China Pharmaceutical University, Nanjing 210009, China.
† Footnotes relating to the title and/or authors should appear here.
Electronic Supplementary Information (ESI) available: [details of any supplementary information available should be included here]. See DOI: 10.1039/x0xx00000x

Please do not adjust margins
